# Exploring the Catalytic Mechanisms of a Newly Identified Salt-Activated Alginate Lyase from *Pseudoalteromonas carrageenovora* ASY5

**DOI:** 10.3390/md23060254

**Published:** 2025-06-15

**Authors:** Xiaoyan Zhuang, Chao Jiao, Zewang Guo, Qiong Xiao, Jun Chen, Fuquan Chen, Qiuming Yang, Yi Ru, Huifen Weng, Siyuan Wang, Anfeng Xiao, Yonghui Zhang

**Affiliations:** 1 College of Ocean Food and Biological Engineering, Jimei University, Xiamen 361005, China; zxy@jmu.edu.cn (X.Z.); guozw1990@jmu.edu.cn (Z.G.); xiaoqiong129@jmu.edu.cn (Q.X.); chenjun@jmu.edu.cn (J.C.); fqchenhy0109@jmu.edu.cn (F.C.); yangqm@jmu.edu.cn (Q.Y.); 201971000023@jmu.edu.cn (Y.R.); wenghuifen@jmu.edu.cn (H.W.); 2 Suzhou Institute for Drug Control, Suzhou 215000, China; wangsiyuan880923@163.com

**Keywords:** alginate lyase, heterologous expression, salt-activated, antioxidant activity

## Abstract

Alginate lyases are critical enzymes in hydrolyzing alginate into alginate oligosaccharides (AOS), which are bioactive compounds known for their antioxidant properties and ability to lower serum glucose and lipid concentrations. However, elucidating catalytic mechanisms and discovering enzymes with enhanced catalytic efficiency remain long-term challenges. Here, we report AlgL2491, a novel bifunctional and cold-adapted alginate lyase from *Pseudoalteromonas carrageenovora* ASY5, belonging to the polysaccharide lyase family 18. This enzyme uniquely cleaves both polyguluronic (polyG) and polymannuronic (polyM), predominantly releasing disaccharides, trisaccharides, and tetrasaccharides after 12 h of hydrolysis. The enzyme achieves peak catalytic efficiency at 35 °C and pH 7.5, with activity increasing 5.5-fold in 0.5 M of NaCl. Molecular dynamics simulations demonstrate that salt ions enhance structural stability by minimizing conformational fluctuations and strengthening interdomain interactions, providing mechanistic insights into its salt-activated behavior. The alginate oligosaccharides (AOS) exhibit excellent free radical-scavenging activities of 86.79 ± 0.31%, 83.42 ± 0.18%, and 71.28 ± 2.27% toward hydroxyl, ABTS, and DPPH radicals, with IC50 values of 8.8, 6.74, and 9.71 mg/mL, respectively. These findings not only reveal the salt-activation mechanism of AlgL2491 and highlight the potential value of its hydrolysate in antioxidant activity but also provide a sustainable industrial solution in industrial-scale AOS production directly from marine biomass, eliminating the need for energy-intensive desalination of alginate, which may inform future biocatalyst design for marine polysaccharide valorization.

## 1. Introduction

The global seaweed market is projected to expand by USD 5.56 billion, with an estimated compound annual growth rate (CAGR) of 7.22% from 2023 to 2028, according to data from Technavio Plus [1]. Alginate, an anionic linear polysaccharide primarily extracted from the cell walls of brown algae [2], comprises β-*D*-manuronic acid (M) and α-*L*-guluronic acid (G). It is renowned for its gelation, thickening, stabilization, and metal ion chelation properties, making it extensively utilized in the food [3], medical treatment [4], printing and dyeing [5], and chemical industry fields [6]. However, overcoming difficulties from high molecular weight and poor solubility remains a perennial objective for the application of alginate [7]. Consequently, alginate oligosaccharides (AOS) have garnered increasing interest due to their retention of alginate’s functional properties alongside reduced molecular weight and improved solubility [8]. Notably, research has demonstrated that AOS can induce cytokine production in RAW264.7 cells [9], reduce serum glucose and lipid concentrations [10], and promote the growth of probiotic bacteria such as *Bifidobacterium bifidum*, *Bifidobacterium longum* [11], and Lactobacilli [12].

Alginate lyase, a crucial enzyme for alginate degradation, produces AOS through a β-elimination process that generates a double bond between C-4 and C-5, generating 4-deoxy-L-erythro-hex-4-ene pyranosyluronate at the non-reducing end of the resulting oligosaccharides [13]. A poly-mannuronate (polyM)-preferring alginate lyase, Aly44A, derived from *Bacillus* sp. FJAT-50079, exhibits random endo-acting lyase activity on alginate [14]. Predominantly, alginate lyases isolate from marine-derived organisms, including animals, bacteria, algae, fungi, and viruses [15,16], which vary significantly in their properties depending on the source. Aly35, derived from the marine bacterium *Vibrio* sp. strain H204, contains two catalytic domains of alginate lyase that demonstrate degrading activities for both polyM and polyG [17]. AL2, isolated from *Flammeovirga* sp. strain MY04, exhibits higher hydrolytic activity than that of commercial alginate lyases [18]. Particularly, alginate lyases discovered in bacteria from extreme environments, such as Arctic bacteria or deep-sea hydrothermal vents, exhibit adaptations such as cold adaptation [19] or thermophilicity [20]. Studies have also highlighted salt-activated [21,22,23,24] alginate lyases from marine bacteria, underlining the influence of the high-salt conditions of their native marine environments on their functional characteristics [25,26]. Among these reports, the alginate lyase derived from the marine *Vibrio harveyi* AL-128 exhibited the highest salt activation effect (24-fold increase with 1 M of NaCl) [27].

Despite the extensive development of alginate lyase and the widely recognized salt-regulated properties, the investigation into the salt-activation mechanism of alginate lyase has received scant attention. The mechanism by which salt affects the protein structure of alginate lyase and leads to enzyme activation remains unclear. This gap in knowledge hampers the rational design of alginate lyase to enhance its catalytic performance. Various approaches are employed to explore the mechanism of haloadaptation or halophilicity in enzymes. The most common method involves comparing the DNA [28] and amino acid [29,30] compositions or the protein structures of halophilic enzymes [31] with those of their mesohalophilic counterparts to identify sequence patterns or structural features that explain halophilicity. Other techniques, such as mutagenesis [32], CD/fluorescence spectroscopy [33], and nuclear magnetic resonance [30], have also been employed to study halophilic mechanisms. However, as an intrinsic property of the enzyme, protein motion and flexibility are crucial for catalytic reactions [34,35,36,37]. The aforementioned methods primarily focus on sequence information or transient structural properties, which fail to capture the conformational dynamics induced by the salt environment.

*P. carrageenovora* ASY5 strain, isolated from decomposed kelp in the mangrove soils of Xiamen, is an efficient producer of alginate lyases. Alg823, synthesized by *P. carrageenovora* ASY5, excels in degrading alginate due to its pH tolerance and heat resistance [38]. Aly1281, another enzyme from the same strain, is a salt-activated alginate lyase that primarily releases dimers from alginate [39]. In this study, we characterized a novel cold-adapted PL18 alginate lyase AlgL2491 derived from *P. carrageenovora* ASY5, which proved to be a bifunctional enzyme capable of hydrolyzing both polyG and polyM blocks of alginate. This enzyme also functions as a salt-activated endo-enzyme, predominantly liberating dimers and trimers from sodium alginate. Comparative molecular dynamics studies were conducted to more directly analyze the conformational dynamic changes induced by varying salt concentrations. These studies assessed the conformational dynamics of AlgL2491 in different salt conditions and identified key protein domains critical to the enzyme’s salt-activation mechanism.

## 2. Results

### 2.1. AlgL2491 Sequence Information

**Figure 1:**
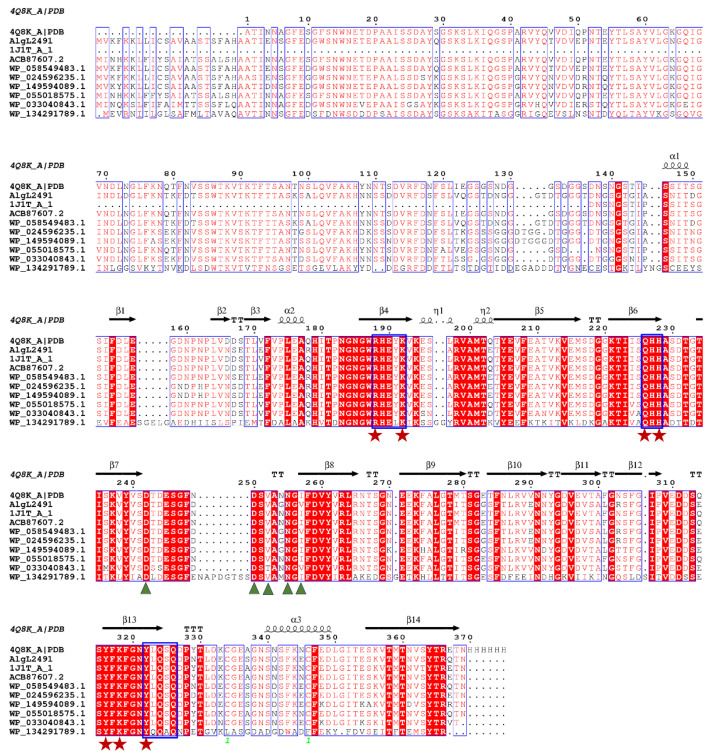
Multiple sequence alignments of AlgL2491 with other alginate lyases were deposited in the NCBI database. The conserved regions were highlighted with a green border. Green triangles indicate the residues involved in the Ca^2+^ binding site. The catalytic sites are marked with red stars.

**Figure 2:**
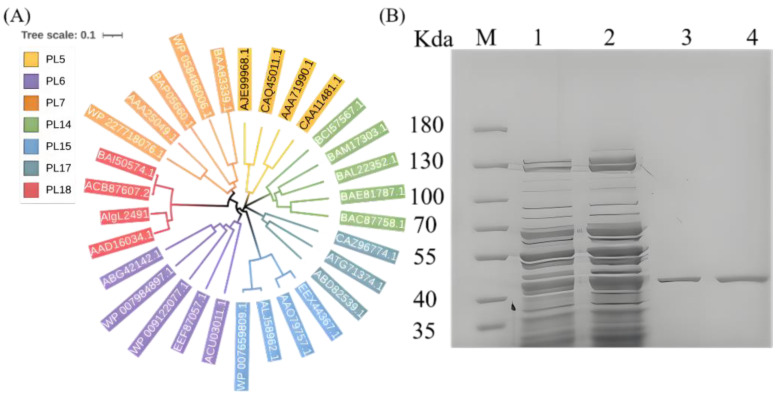
The phylogenetic tree and SDS-PAGE analysis. (**A**) phylogenetic tree of AlgL2491. (**B**) SDS-PAGE of recombinant expression and purification of the seaweed polysaccharide lyase gene AlgL2491. M lane is a protein Marker, lane 1 was the expression of pET-28a-AlgL2491 in *E. coli* BL21. Lane 2 was the expression of the recombinant vector pET-28a-AlgL2491 in *E. coli* BL21 (final concentration of IPTG was 0.05 mmol/L), lane 3 and lane 4 were the target protein AlgL2491 (42 kDa) obtained from the purification of Ni-NTA. AlgL2491, AAA25049.1: *Klebsiella pneumoniae*, AAD16034.1: *Pseudoalteromonas distincta*, BAA83339.1: *Corynebacterium* sp. ALY-1, AAO79757.1: *Bacteroides thetaiotaomicron* VPI-5482, BAC87758.1: *Haliotis discus hannai*, BAE81787.1: *Haliotis discus hannai*, ABD82539.1: *Saccharophagus degradans* 2–40, ABG42142.1: *Paraglaciecola* sp. T6c, CAQ45011.1: *Stenotrophomonas maltophilia* K279a, EEF87057.1: *Bacteroides cellulosilyticus* DSM 14838, ACU03011.1: *Pedobacter heparinus* DSM 2366, EEX44367.1: *Bacteroides finegoldii* DSM 17565, BAI50574.1: *Pseudoalteromonas atlantica*, CAZ96774.1: *Zobellia galactanivorans*, BAL22352.1: *Littorina brevicula*, BAM17303.1: *Littorina brevicula*, WP_007659809.1: *Bacteroides*, WP_007984897.1: *Paraglaciecola chathamensis*, WP_009122077.1: *Bacteroides*, ACB87607.2: *Pseudoalteromonas* sp. SM0524, BAP05660.1: *Flavobacterium* sp. UMI-01, AJE99968.1: *Pandoraea apista*, ALJ58962.1: *Bacteroides cellulosilyticus*, WP_058486006.1: *Defluviitalea phaphyphila*, ATG71374.1: *Microbulbifer* sp. ALW1, BCI57567.1: *Mizuhopecten yessoensis,* WP_227718076.1: *Microbulbifer* sp. Q7, CAA11481.1: *Azotobacter chroococcum*, AAA71990.1: *Pseudomonas aeruginosa*.

The alginate lyase gene comprises 1182 base pairs whose open reading frame (ORF) encodes 393 amino acids (Figure 1, Table S1). The mature enzyme displays a calculated molecular mass of 42.34 kDa and an isoelectric point (pI) of 4.48. Sequence alignment suggests that AlgL2491 is a member of the polysaccharide lyase (PL) family 18 (Figure 2A). Notably, the PL7 alginate lyases consist of three conserved motifs—(RXEXR), (YXKAGXYXQ), and (QXH), which form the active center and are essential for substrate recognition and catalysis [40]. Similarly, PL18 alginate lyases, including AlgL2491, feature equivalent conserved motifs. As depicted in Figure 1, these motifs are RHEYK, YFKFGNYLQ (SA3), and QHH. Moreover, based on the previous research on the alginate lyase aly-SJ02 (PDB ID: 4Q8K-A) from *Pseudoalteromonas* sp. SM0524, we predicted the catalytic site (R190, K194, Q228, H230, Y318, Y324, and K320) and the calcium-binding sites (D244, D252, V254, N257, and V259) in AlgL2491 [41].

### 2.2. Expression and Purification of Recombinant Enzyme AlgL2491

The recombinant strain BL21 (DE3)/pET28a-AlgL2491 was induced with IPTG to express the enzyme AlgL2491. Following induction, the supernatant was collected via centrifugation to assess the purity of alginate lyase using SDS-PAGE. The IPTG-induced supernatant revealed a distinct protein band at 42 kD (Figure 2B), aligning with the predicted molecular weight of 42.34 kD. This result confirms the successful expression of the recombinant alginate lyase gene AlgL2491 in *Escherichia coli*. Both induced and uninduced fermentation supernatants were subsequently purified using a nickel column, yielding a single protein band, which indicates that the recombinant enzyme AlgL2491 was successfully purified.

### 2.3. Biochemical Characterization of AlgL2491

**Figure 3:**
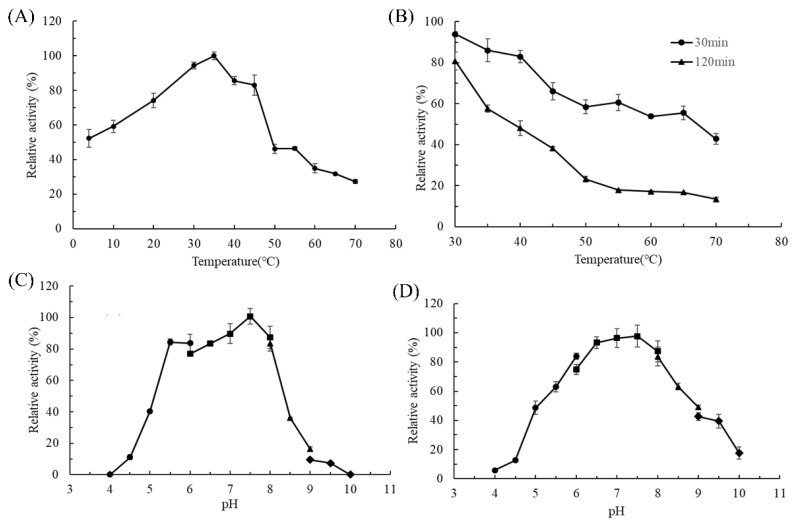
Effects of pH and temperature on the activities and stabilities of AlgL2491. (**A**) The optimal temperatures of the enzymes were determined by measuring the activity at various temperatures (4–70 °C). (**B**) The thermostabilities of AlgL2491. The residual activity was determined at the optimal temperatures after incubation at various temperatures (30–70 °C) for 30 min or 2 h. (**C**) The optimal pH values of AlgL2491 were determined by measuring the activity in the 0.05 mol/L of acetic acid–sodium acetate buffer (●: pH 4.0–6.0), 0.05 mol/L of Na_2_HPO_4_-NaH_2_PO_4_ buffer (■: pH 6.0–8.0), and 0.05 mol/L of Tris-HCl buffer (▲: pH 8.0–9.0) and 0.05 mol/L of glycine–sodium hydroxide buffer (◆: pH 9.0–10.0). (**D**) The pH stabilities of AlgL2491. The residual activity was measured after the enzyme was incubated in the pH range of 4.0–10 with the above buffers for 24 h at 4 °C.

The impact of temperature on the activity of AlgL2491 is depicted in Figure 3A, revealing that optimum enzyme activity is achieved at 35 °C. Within a temperature range of 4 to 45 °C, AlgL2491 maintains activities exceeding 50% of its optimum performance. As illustrated in Figure 3B, the thermostability of AlgL2491 was assessed over temperatures from 30 to 70 °C, among which AlgL2491 retained 94% of its initial activity at 30 °C and 43% at 70 °C after a 30-min incubation. Many marine-derived enzymes tend to deactivate under low-temperature conditions [42], while cold-adapted enzymes often exhibit lower optimal temperatures, enhanced activity at reduced temperatures, and decreased thermostability relative to their mesophilic counterparts. AlgL2491, with an optimal temperature of 35 °C, retains 52% of its maximal activity at 4 °C and becomes rapidly inactivated at temperatures exceeding 45 °C. These findings suggest that AlgL2491 possesses cold-adapted characteristics.

Cold-adapted alginate lyases exhibit two primary characteristics. Firstly, these enzymes operate optimally at relatively low temperatures, typically not exceeding 40 °C, while still maintaining high catalytic activity. Secondly, they display limited temperature stability; enzyme activity sharply declines or may become inactivated entirely when temperatures rise above 30 °C, indicating poor thermal stability [43]. AlgL2491 had a low optimal temperature of 35 °C, retained 52% of its highest activity at 4 °C, and rapidly inactivated at temperatures higher than 45 °C. This result suggests that AlgL2491 has cold-adapted characteristics.

The optimal reaction pH for AlgL2491 was evaluated by measuring its enzymatic activities in buffers with pH values ranging from 4.0 to 10.0. As illustrated in Figure 3C, the enzyme’s optimal pH is 7.5, where AlgL2491 maintains over 75% of its optimum activity at mildly acidic to slightly alkaline conditions (pH 5.5 to 8). The activity of AlgL2491 is virtually undetectable below pH 4.5, demonstrating its high sensitivity to acidic conditions. These findings are consistent with those of most marine-derived alginate lyases, which typically exhibit optimal pH levels between 6.0 and 9.0 [44,45,46]. The pH stability of AlgL2491 was also assessed by evaluating its residual activity (Figure 3D) after 24 h of incubation at 4 °C in various pH buffers (4.0–10.0). AlgL2491 was most stable at pH 7.5 and could retain over 75% and 50% of its activities within pH ranges of 6.0 to 8.0 and 5.0 to 9.0, respectively.

### 2.4. Salt Stabilization of AlgL2491

**Figure 4:**
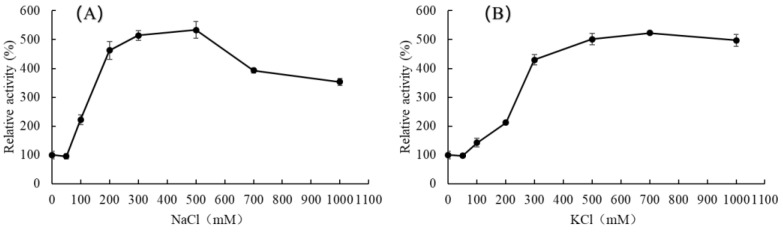
Effects of (**A**) NaCl and (**B**) KCl concentration on the enzymatic activity of AlgL2491. AlgL2491 activities were measured by using standard conditions as described in Section 3. The activity observed without salt addition was considered to be 100% for calculations of relative activities. ● represents the relative activity average value of three replicates. Data represent the mean ± standard deviation of triplicate measurements.

Due to the unique marine environment where specific bacteria proliferate and evolve, enzymes derived from these marine bacteria often demonstrate exceptional enzymatic properties such as salt tolerance and salt activation. Figure 4 illustrates the impact of varying salt concentrations, including NaCl and KCl, on enzyme activities. The enzyme activity of AlgL2491 peaked with the addition of 500 mM of NaCl and 700 mM of KCl, improving activity 5.33-fold and 5.23-fold, respectively, compared with that without salt. However, the activity of AlgL2491 increased sharply with salt concentrations below 500 mM for both NaCl and KCl. However, enzyme activity decreased when the salt concentration exceeded a certain value (500 mM for NaCl and 700 mM KCl), suggesting that an optimal salt concentration can significantly enhance AlgL2491 activity. Remarkably, the catalytic efficiency of AlgL2491 was highly responsive around the average oceanic salt concentration (approximately 430 mM), rendering it viable for industrial processing of brown seaweed without necessitating an intensive desalination process [47]. It was further demonstrated that alginate lyases from different sources exhibit varying levels of salt activation and optimal salt concentrations. For instance, AlgNJU-03 from *Vibrio* sp. NJU-03 showed no reaction to Na^+^ or K^+^ [48], whereas Algb from *Vibrio* sp. W13’s activity increased 2.2-fold with 0.3 M of NaCl and Alginate lyase from *Microbulbifer* sp. ALW1 increased 5.1-fold with 0.5 M of NaCl [49]. Among the most notable examples was the alginate lyase from *Vibrio harveyi*-28, which exhibited a 24-fold increase at 1 M of NaCl. In this study, the activity of AlgL2491 from *P. carrageenovora* ASY5 increased 5.33-fold and 5.23-fold at 0.5 M of NaCl and 0.7 M of KCl, respectively.

**Figure 5:**
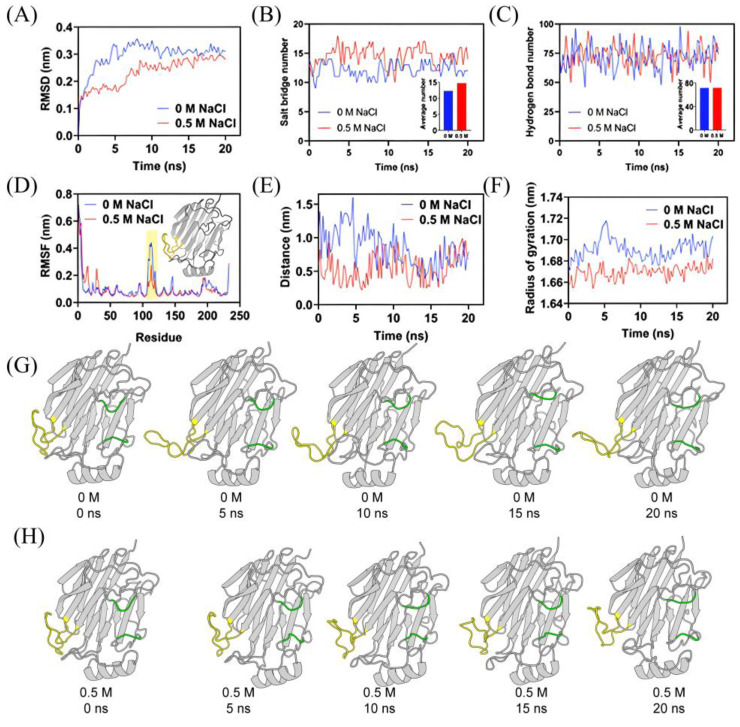
The effect of NaCl and KCl on enzymatic activity of AlgL2491. (**A**) RMSDs of AlgL2491 in 0 mM and 0.5 mM of NaCl during 20 ns MD simulations as a function of time relative to its initial structure. (**B**) RMSFs of AlgL2491 in 0 mM and 0.5 mM of NaCl during 250 ns MD simulations. (**C**) The gyrate radius of AlgL2491 in 0 mM and 0.5 mM of NaCl during 20 ns MD simulations. (**D**) The distance of AlgL2491 with 0 mM and 0.5 mM of NaCl. (**E**) The cartoon presentation of AlgL2491 with 0 mM of NaCl. (**F**) The cartoon presentation of AlgL2491 with 0.5 mM of NaCl. The activity without adding metal ions was relatively taken as 100%. Each value represents the mean of three replicates ± standard deviation. (**G**,**H**) Structural analysis of AlgL2491 in 0 mM and 5 mM of NaCl during 20 ns MD simulations.

To explore the effect of salt ions on alginate lyase, dynamic simulations were performed on Alg2491 to observe structural changes under varying NaCl concentrations. Molecular dynamics (MD) simulations were utilized to examine the influence of 0 mM and 500 mM of NaCl on AlgL2491 at 35 °C. By referencing the initial conformation, we measured the root mean square deviation (RMSD) values over a 20 ns simulation period, depicted in Figure 5A. After adding 500 mM of NaCl, the stable average RMSD of AlgL2491 was approximately 2.832 Å, compared to 3.101 Å without NaCl. The consistently lower RMSD values under 500 mM of NaCl throughout the simulation demonstrate that salt ions can significantly enhance the structural stability of AlgL2491 through their interactions. Figure 5B,C illustrates that different protein structure domains in AlgL2491 respond variably to salt addition, which increases the enzyme’s structural compactness and improves stability. As indicated in Figure 5D, after introducing salt ions, the salt bridge distance within the alginate lyase structure remained close to 6 Å until 10 ns, beyond which it stabilized, unlike the notable fluctuations observed without salt ions.

After the analysis of the weak interactions of protein internal salt bridges and hydrogen bonds, we found that the addition of salt ions exhibited effects not on the number of internal hydrogen bonds (Figure 5C) but on the average number of internal salt bridges formed by AlgL2491 that increased from 12.3 to 14.7 (Figure 5C), indicating that salt bridges contribute to structural stability at high salt concentrations. Meanwhile, the flexibility of the loop region (S105-D123) located at the entrance side of the catalytic cavity significantly decreased under the influence of salt (Figure 5D), which might affect substrate binding or product release, thereby affecting the enzyme’s catalytic ability. The lid loop is a crucial structure affecting substrate binding during the catalytic process of AlgL2491 [39]. The distance between the lid loops significantly decreased at the beginning of the simulation with 0.5 M of salt ions (Figure 5E), which might facilitate the binding of alginate [39]. Furthermore, when we analyzed the structural changes during the simulation process, as shown in Figure 5G,H, the loose loop region (S105-D123) transformed into a compact structure under 0.5 M of salt ions, which was also reflected in the differentiation in gyration radius (Figure 5F).

### 2.5. Substrate Specificity and Mode of Enzyme Action of AlgL2491

The substrate specificity of alginate lyase AlgL2491 was investigated using three different substrates, as delineated in Table 1. The results reveal that AlgL2491 possesses broad substrate specificity and functions as a bifunctional enzyme, capable of hydrolyzing both polyG and polyM. Compared to other alginic acid lyases derived from various marine *Pseudomonas* species, AlgL2491 demonstrates remarkably broader specificity. These outstanding characteristics suggest potential applications of this enzyme in industrial production. Similar observations were reported for the alginate lyase from *Streptomyces armeniacus* [20]. In contrast, as documented in the literature, certain alginate lyases exhibit specificity solely for polyM or polyG [16].

**Figure 6:**
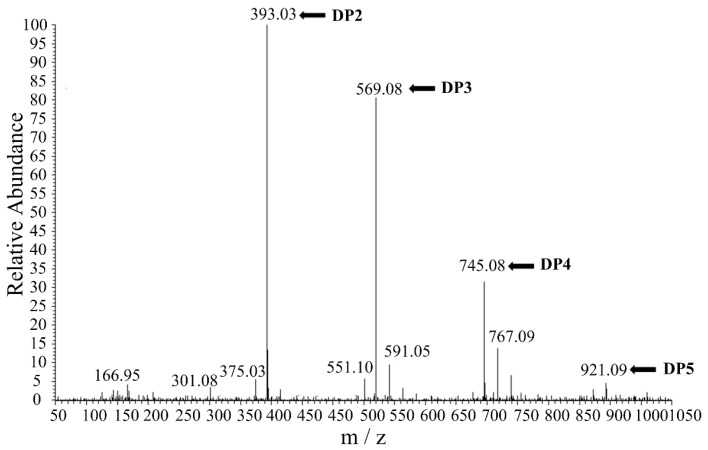
ESI-MS analysis of the hydrolysis products of AlgL2491 with alginate sodium. DP: Degrees of polymerization.

After 12 h of hydrolysis, the degradation products of alginate lyase AlgL2491 were analyzed using ESI-MS (Figure 6). Disaccharides, trisaccharides, and tetrasaccharides comprised the majority of the alginate hydrolysates, indicating that AlgL2491 is an endo-type alginate lyase [50]. This enzyme could potentially facilitate the production of alginate oligosaccharides (AOS). The hydrolysates primarily consisted of oligomers with degrees of polymerization (DP) ranging from 2 to 5, similar to other alginate lyases like Algb from the marine bacterium *Vibrio* sp. W13 [26] and AlgNJ-07 from *Serratia marcescens* NJ-07 [3]. Various studies demonstrate that low molecular weight AOS rich in sulfuric acid substantially inhibits platelet aggregation induced by methoxinic acid and collagen in rats, thereby reducing the risk of venous and arterial thrombosis [51]. Yamamoto et al. have confirmed AOS’s antitumor properties [9], while Kawada et al. reported that dimers, trimers, and tetramers, particularly those with guluronic acid at the reducing end, effectively induce keratinocyte proliferation in the presence of epidermal growth factor [52]. As a key enzyme in alginate degradation, particularly against polyM and polyG, alginate lyase presents substantial industrial utility. AlgL2491 efficiently degrades alginate into dimers, trimers, and tetramers and could thus be instrumental in producing specific AOS from alginate.

### 2.6. Antioxidant Function of the Hydrolysates of AlgL2491

**Figure 7:**
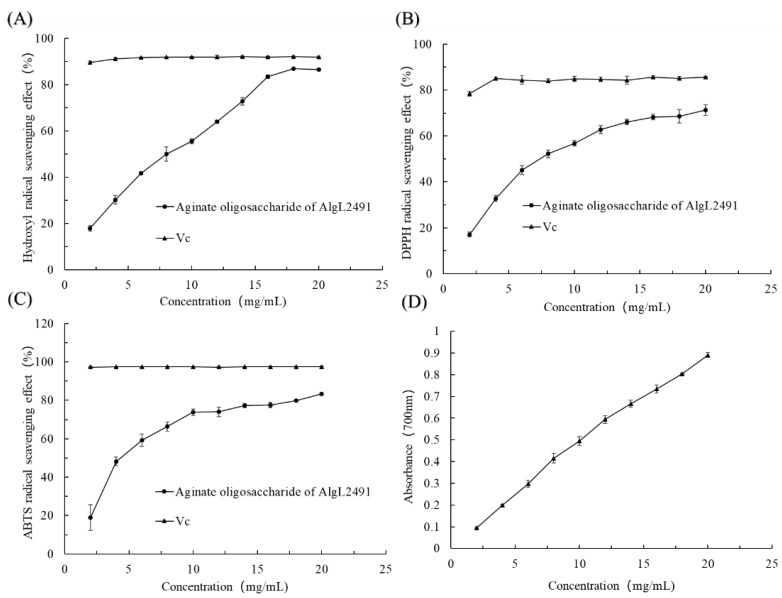
Antioxidant function of alginate oligosaccharides. (**A**) Scavenging effect on hydroxyl radicals. (**B**) Scavenging effect on DPPH radicals. Data represent the mean ± standard deviation of triplicate measurements. (**C**) Scavenging effect on ABTS radicals. (**D**) Reducing ability. Vc: vitamin C.

Several methods were employed to evaluate the antioxidant activity of alginate oligosaccharides (AOS), such as hydroxyl radical scavenging, DPPH and ABTS radical-scavenging assays, and ferric reducing assays. Hydroxyl radicals, the most reactive species, can induce lipid peroxidation in unsaturated fatty acids, damaging membrane structures and functions, and potentially causing cell necrosis or mutation [53]. Therefore, mitigating hydroxyl radicals is crucial for protecting living organisms. In this study, the hydroxyl radical-scavenging capacity of AOS was determined using salicylic acid as a molecular probe, demonstrating a concentration-dependent scavenging ability that peaked at 86.79 ± 0.31% at an 18.0 mg/mL concentration (Figure 7A). Additionally, the IC50 of the hydrolysates was 8.8 mg/mL. In this research, DPPH and ABTS radicals served as substrates to gauge AOS’s antioxidant efficacy. At a concentration of 20 mg/mL, AOS exhibited optimal scavenging activities of 83.42 ± 0.18% for ABTS+ and 71.28 ± 2.27% for DPPH radicals. The IC50 values for ABTS+ and DPPH were 6.74 mg/mL and 9.71 mg/mL, respectively, comparable to those of other alginate oligosaccharides reported by Zhang et al. [39], who noted that these oligosaccharides primarily consisted of low-molecular-weight AOS (di-alginate oligosaccharide), enhancing their radical-scavenging activity [9].

Reducing power is a key index for evaluating antioxidant activity; the greater reducing capacity proves the stronger antioxidant capacity [54]. The determination results of reducing power are shown in Figure 7D; the reducing power improved with the increase in concentration, displaying a linear correlation, while the reducing effects of AOS increased from 0.094 to 0.890 at 700 nm with increasing enzyme concentration. The results show that AOS has some reducibility.

There are reports that the antioxidant capacity of AOS mainly originates from a conjugated alkene acid structure [46]. Some researchers also proposed the catalytic mechanism of alginate lyase for the formation of the alkene acid structure based on structural and mutational analysis. In general, the AOS obtained from AlgL2491 presents good scavenging activities toward hydroxyl, ABTS, and DPPH radicals, respectively. Alginate oligosaccharides have good antioxidant activity and might be exploited as available antioxidant products in the food and pharmaceutical industries.

## 3. Materials and Methods

### 3.1. Strains and Plasmids

The *P. carrageenovora* ASY5 strain was screened from Xiamen mangrove soil (preserved in China Industrial Microbial Culture Collection Management Center, deposit number: CICC 23819). *E. coli* BL21 (DE3) and pET-28a (+) were preserved in the Key Laboratory of Food Microbiology and Enzyme Engineering of Fujian Province (Jimei University, Xiamen, China).

### 3.2. Construction of Recombinant E. coli

The AlgL2491 gene from the genomic DNA of *P. carrageenovora* ASY5 was used for primer design for PCR amplification. The forward primer was 5ʹ- CCGGAATTCATGGTTAAATTTAAAAAGTT-3ʹ and the reverse primer was 5ʹ- CCCAAGCTTGTTTGTCTGACGGGTATAAC-3ʹ. The Aly gene was obtained by PCR amplification using the genomic DNA of *P. carrageenovora* ASY5 as the template. The PCR products were analyzed by agarose gel electrophoresis, then purified and digested with *EcoR* I and *Hind* III. The recovered Aly gene was ligated with T4 DNA ligase to pET-28a (+) plasmid digested with the same restriction enzymes, which was then transformed into *E. coli* BL21 (DE3) competent cells for enzyme expression.

### 3.3. Expression and Purification of AlgL2491

The constructed recombinant *E. coli* containing the AlgL2491 gene was inoculated in the LB medium containing Kanamycin of 50 mg/L at 37 °C with horizontal shaking at 180 rpm, and 0.05 mmol/L IPTG was added for inducing AlgL2491 expression when OD_600_ reached 0.6–0.8. The cell was allowed to grow at 16 °C with horizontal shaking at 180 rpm for 20 h before the cells were collected by centrifugation. The collected cells were sonicated in a lysis buffer (300 mM of NaCl, 15 mM of imidazole, 50 mM of NaH_2_PO_4_, pH7.5), and centrifuged at 8000 rpm for 20 min at 4 °C to obtain crude AlgL2491, which was then loaded on an Ni-NTA agarose column equilibrated using a lysis buffer. The column was first washed with washing buffer (300 mM of NaCl, 15 mM of imidazole, 50 mM of NaH_2_PO_4_, pH7.5) to remove the protein impurities, and the recombinant AlgL2491 was eluted with elution buffer (300 mM of NaCl, 250 mM of imidazole, 50 mM of NaH_2_PO_4_, pH7.5). The eluted fractions with Aly activities were collected, combined and desalted using a Macrosep Advance Centrifugal Device (cut-off 10 kDa, Pall, East Hills, NY, USA), and analyzed by 10% sodium dodecyl sulfate polyacrylamide gel electrophoresis (SDS-PAGE).

### 3.4. Phylogenetic Analyses of the AlgL2491 Sequence

The phylogenetic tree was constructed using the Molecular Evolutionary Genetics Analysis (MEGA, version 11) program, based on the neighbor-joining method. Then, iTOL (https://itol.embl.de/, accessed on 30 May 2025) was used to draw and visualize the phylogenetic tree.

### 3.5. Measurement of AlgL2491 Activity

Exactly 200 μL of purified recombinant enzyme AlgL2491 was mixed with 800 μL of sodium alginate (5 mg/mL, pH 7.5) and allowed to react at 50 °C for 40 min before a boiling water bath terminated the reaction. Then, 1 mL of DNS solution was added and reacted in a boiling water bath for 10 min; the absorbance was measured at 540 nm. One unit of AlgL2491 activity was defined as the amount of enzyme required to catalyze the production of 1 μmol of reducing sugar per minute under the above conditions.

### 3.6. Effects of Temperature and pH on AlgL2491 Activity and Stability

To investigate the effect of temperature on the activity of AlgL2491, the activities of alginate lyase at different temperatures (4–70 °C) were measured under the assay conditions described previously, and the relative enzyme activities were calculated using the activity obtained at the optimum temperature as 100%. To investigate the effect of temperature on the stability of AlgL2491, AlgL2491 was incubated at different temperatures (30–70 °C) for 2 h, and the residual enzyme activities were measured under the assay conditions described previously and calculated using the initial activities as 100%.

To investigate the effect of pH on the activity of AlgL2491, the activities of alginate lyase using different buffers, including 0.05 mol/L of acetic acid–sodium acetate buffer (pH 4.0–6.0), 0.05 mol/L of Na_2_HPO_4_-NaH_2_PO_4_ buffer (pH 6.0–8.0), 0.05 mol/L of Tris-HCl buffer (pH 8.0–9.0), and 0.05 mol/L of glycine–sodium hydroxide buffer (pH 9.0–10.0), were measured under the assay conditions described previously, and the relative enzyme activities were calculated using the activity obtained at the optimum pH as 100%. To investigate the effect of pH on the stability of AlgL2491, AlgL2491 was incubated in the above-mentioned buffers (pH 4.0–10.0) at 4 °C for 24 h, and the residual enzyme activities were measured under the assay conditions described previously and calculated using the highest residual activities as 100%.

### 3.7. Effects of Salts on the Activity and Kinetic Parameters of AlgL2491

The effects of salts on the activity of Aly were measured under the assay conditions described previously, with different concentrations of NaCl or KCl (0–1000 mM), and the relative enzyme activities were calculated using the activity obtained without salts as 100%.

### 3.8. Substrate Specificity of AlgL2491

The substrate specificities of purified AlgL2491 were investigated by measuring Aly activity under the assay conditions described previously using 2 mg/mL of sodium alginate, D-polymannuronic acid (polyM), L-polyguluronic acid (polyG), and alginate oligosaccharide (3.3 kDa) as substrates.

### 3.9. ESI-MS Analysis of the Degradation Products of AlgL2491

For the degradation reaction of AlgL2491, 200 µL of purified enzyme was added to 800 µL of substrate solution (5 mg/mL of sodium alginate). The degradation reaction was allowed to proceed at 50 °C for 12 h. The reducing sugar content in the reaction mixture was determined periodically. When the reducing sugar content reached a stable level, the reaction mixture was boiled for 10 min and centrifuged at 12,000 rpm for 10 min. Three times the volume of ethanol was added to the hydrolysates, and the mixture was incubated at 4 °C for 2 h. After incubation, the supernatant was concentrated by rotary evaporation and lyophilized.

The composition and degree of polymerization (DP) of the products were further determined by ESI-MS. Then, 2 µL of degradation products was loop-injected to electrospray ionization mass spectrometry (ESI–MS) (Bruker Esquire HCT, Billerica, MA, USA) in a negative-ion mode with the following settings: BEH C18(150 × 2.1 mm, 1.7 μm); Calibration Dynamic 2; Capillary (4.00 kV); Cone (20.00 V); Source Temperature (150 °C); Desolvation Temperature (350 °C); Cone Gas Flow (50 L/h); Desolvation Gas Flow (500 L/h).

### 3.10. Antioxidant Function of the Alginate Degradation Products of AlgL2491

#### 3.10.1. Scavenging Activity of Hydroxyl Radical

Hydroxyl radical-scavenging activity was determined in accordance with a previously described method [39]. Briefly, 0.1 mL of FeSO_4_ (9.0 mM), 0.6 mL of deionized water, 2 mL of oligosaccharide solution, 0.1 mL of H_2_O_2_ (8.8 mM), and 0.1 mL of ethanol salicylate (9.0 mM) were mixed and incubated at 37 °C for 30 min. The absorbance of the mixture was then determined at 510 nm. Distilled water and vitamin C (Vc) were used as the blank and positive control, respectively. Scavenging activity (%) was calculated by using the following equation:

Hydroxyl free-radical scavenging activity (%) = (A0 − Asample)/A0 × 100(1)

where A0 and Asample are the absorbance of the blank and final absorbance of each sample at 510 nm, respectively.

#### 3.10.2. Scavenging Activity of 2,2-Diphenyl-1-Picrylhydrazyl (DPPH)

DPPH radical-scavenging activity was determined by using a previous method with minor modifications [55]. Oligosaccharide solutions (500 μL) of different concentrations were mixed with DPPH reagent (500 μL) and then incubated in the dark at room temperature for 30 min. The absorbance of the mixture was determined at 517 nm. Distilled water was set as the blank, and Vc was used as the positive control. The scavenging activity (%) of DPPH was calculated by using the following equation:

DPPH radical scavenging activity (%) = (A0 − Asample)/A0 × 100(2)

where A0 and Asample are the absorbance of the blank and final absorbance of each sample at 517 nm, respectively.

#### 3.10.3. Scavenging Activity of 2,2′-Azinobis-(3-Ethylbenzthiazoline-6-Sulphonate) (ABTS)

ABTS radical-scavenging activity was determined according to a previous method with minor modifications [56]. ABTS (7 mM) and K_2_S_2_O_8_ (2.45 mM) solutions were mixed at equal volumes and stored in the dark for 16 h to produce ABTS radicals (ABTS+). The mixture was diluted with PBS (pH 7.0) to an absorbance of A734 nm = 0.700 ± 0.020. Then, 0.1 mL of oligosaccharide solution and 1 mL of ABTS solution were mixed and incubated at 37 °C for 15 min. The absorbance of this solution was measured at 734 nm. Distilled water was set as the blank, and Vc was used as the positive control. The scavenging activity (%) of ABTS was calculated by using the following equation:

ABTS radical scavenging activity (%) = (A0 − Asample)/A0 × 100(3)

where A0 and Asample are the absorbance of the blank and final absorbance of each sample at 734 nm, respectively.

#### 3.10.4. Ferric Reducing Power

Ferric reducing power was determined in accordance with a previously described method [57] with slight modifications. Briefly, 300 μL of degradation products of different concentrations was mixed with 350 μL of 0.2 M sodium phosphate buffer (pH 7.0) and 350 μL 10 g/L of [K_3_Fe(CN)_6_]. After 20 min of incubation at 50 °C, the mixture was added to a mixture of 350 μL 100 g/L of trichloroacetic acid and 150 μL 10 g/L of FeCl_3_. The absorbance of the mixture was measured at 700 nm by using a UV spectrophotometer. Distilled water was used as the blank.

### 3.11. Protein Simulation

The structure of AlgL2491 was constructed using the I-TASSER server (http://zhanglab.ccmb.med.umich.edu/I-TASSER, accessed on 1 January 2025) with Alginate lyase from *Alteromonas* sp. 272 (PDB ID: 1j1tA) [58], and AlyA1 from *Zobellia galactanivorans* (PDB ID: 3zpy) as threading templates.

## 4. Conclusions

A gene encoding a novel alginate lyase, AlgL2491, was identified and cloned from *P. carrageenovora*. The recombinant enzyme was subsequently purified using Ni-NTA agarose and characterized. It has a molecular weight of approximately 42.34 kDa and demonstrates peak activity towards sodium alginate at 35 °C and pH 7.5. AlgL2491 retains over 75% of its activity within a pH range of 6.0–8.0, showcasing excellent pH stability. Moreover, enzyme activity increased 5.5-fold upon the addition of NaCl to a final concentration of 0.5 M. ESI-MS analysis indicated that AlgL2491 predominantly releases disaccharides, trisaccharides, and tetrasaccharides. Therefore, this enzyme could serve as an effective tool for the preparation of alginate oligosaccharides with a low degree of polymerization.

## Figures and Tables

**Table 1:** Substrate specificity of AlgL2491. Enzyme activity against the substrates at 35 °C and pH 7.5 was determined spectrophotometrically at 235 nm. Values are mean ± standard deviation from three independent experiments.

Substrate	Relative Activity (%)
alginate	100.00 ± 5.41
polyM	83.86 ± 0.41
polyG	90.42 ±1.16

## Data Availability

The original contributions presented in this study are included in the article. Further inquiries can be directed to the corresponding authors.

## Supplementary Materials

The following supporting information can be downloaded at: https://www.mdpi.com/article/10.3390/md23060254/s1, Table S1: The amino acid sequence of AlgL2491 from *P. carrageenovora* ASY5.
