# Supplementary material for: Exploring the Catalytic Mechanisms of a Newly Identified Salt-Activated Alginate Lyase from Pseudoalteromonas carrageenovora ASY5
Source: Mar Drugs. 2025 Jun 15;23(6):254. doi: 10.3390/md23060254 (PMC12193947; doi:10.3390/md23060254)
Supplement: Supplementary file 1 [file marinedrugs-23-00254-s001.zip › marinedrugs-3685877-supplementary.pdf]

Supplementary Materials

Exploring the Catalytic Mechanisms of a Newly Identified Salt-Activated Alginate Lyase from *Pseudoalteromonas. carrageenovora* ASY5

Xiaoyan Zhuang [1], Chao Jiao [1], Zewang Guo [1], Qiong Xiao [1], Jun Chen [1], Fuquan Chen [1], Qiuming Yang [1], Yi Ru [1], Huifen Weng [1], Siyuan Wang [2], Anfeng Xiao [1]* and Yonghui Zhang [1]*

Affiliations:

1 College of Ocean Food and Biological Engineering, Jimei University, Xiamen, 361005, P. R. China.

2 Suzhou Institute for Drug Control, Suzhou, 215000, P. R. China.

* Correspondence: xxaaffeng@jmu.edu.cn (A. Xiao), yhz@jmu.edu.cn (Y. Zhang)

Table S1: The amino acid sequence of AlgL2491 from *P. carrageenovora* ASY5.

ATGGTTAAATTTAAAAAGTTACTTATTTGCTCTGCAGTTGCTGCAAGCACTTCTTTTGCG

CACGCTGCAACGATTGAAAATTCAGGTTTTGAAGATGGCTGGAGTAATTGGAATGAAA

CAGAGCCTGCTGCGCTATCTTCAGATGCATACCAAGGTTCAAAGTCTTTAAAAATTCAA

GGTAGCCCGGCCCGTGTTTATCAAACTGTGGATGTTGAGCCAAATACAGAGTACACATT

GAGTGCGTATGTGCTGGGTAATGGTCAAATTGGTATCAATGATTTAGATGGCTTATTTAA

AAATACTAAATTTGATACGTCTTCTTGGACCAAAGTAACTAAAACTTTTACTACAGCAA

GTAAAAGTGCGCTTCAAGTATTTGCTAAGCATAATAATAGTTCTGATGATGTTCGATTTG

ATTCATTTTCTTTAGTGCAAGGTAGTACTGATAACGGTGGTGGCACTGACACTGGTGGC

GGGACGGATAATGGCAGCGGTACCGGTATAGCAAGTAATATTACTGATGGCAGTATTTT

TGATCTCGAAGGGGATAACCCTAACCCATTAGTAAATAGTGAAACTCTAGAGTTTGTTC

CTTTGGAGGCTCGCCATATTACTCCAAATGGAAATGGCTGGCGTCATGAATATAAAGTT

AAAGAAAGTGCACGCGTTGCAATGACTGAGACCTATGAAGTTTTTGAAGCCACTGTCA

AAGTTGAAATGTCTGATGGCGGAAAAACAATTATTTCGCAGCATCATGCTAGCGATACT

GGAACAATCTCTAAAGTGTATGTTTCAGATACCGATGAATCTGGGTTTGATGACAGCGT

AGCTGGAAATGGCGTTTTTGACGTTTATGTTCGTCTGCGTAACACCAGCGGTAATGAAG

AAAAATTTGCCCTTGGTACTATCACTAGTGGTGGCTCGTTTACTCTTCGTGTTGAAAATA

ACTATGGCGATGTTGATGTTAGCGCACTAGGTCGCTCATTTGGTATACCTGTAGAAGATG

ACTCAGAATCATACTTTAAATTTGGTAATTACCTGCAATCACAAGACCCTAATACATTAG

ATGAGTGCGGGGAATCTGGTAATTCGGATTCTTTCAAAGAGTGCTTTGAAGATTTAGGG

ATTACAGAGTCTAAAGTAACAATGACGAATGTTAGTTATACCCGTCAGACAAACTAA
